# Potential role of glutathione in evolution of thiol-based redox signaling sites in proteins

**DOI:** 10.3389/fphar.2015.00001

**Published:** 2015-03-10

**Authors:** Kaavya A. Mohanasundaram, Naomi L. Haworth, Mani P. Grover, Tamsyn M. Crowley, Andrzej Goscinski, Merridee A. Wouters

**Affiliations:** ^1^School of Medicine, Faculty of Health, Deakin UniversityGeelong, VIC, Australia; ^2^School of Life and Environmental Sciences, Faculty of Science, Engineering and the Built Environment, Deakin UniversityGeelong, VIC, Australia; ^3^Australian Animal Health Laboratory, Animal, Food and Health Sciences Division, Commonwealth Scientific and Industrial Research OrganisationGeelong, VIC, Australia; ^4^School of Information Technology, Faculty of Science, Engineering and Built Environment, Deakin UniversityGeelong, VIC, Australia

**Keywords:** cross-strand disulfide, forbidden disulfide, redox-active disulfide, exaptation, disulfide evolution, CD4 evolution, AKT evolution, post-translational cysteine modification

## Abstract

Cysteine is susceptible to a variety of modifications by reactive oxygen and nitrogen oxide species, including glutathionylation; and when two cysteines are involved, disulfide formation. Glutathione-cysteine adducts may be removed from proteins by glutaredoxin, whereas disulfides may be reduced by thioredoxin. Glutaredoxin is homologous to the disulfide-reducing thioredoxin and shares similar binding modes of the protein substrate. The evolution of these systems is not well characterized. When a single Cys is present in a protein, conjugation of the redox buffer glutathione may induce conformational changes, resulting in a simple redox switch that effects a signaling cascade. If a second cysteine is introduced into the sequence, the potential for disulfide formation exists. In favorable protein contexts, a bistable redox switch may be formed. Because of glutaredoxin's similarities to thioredoxin, the mutated protein may be immediately exapted into the thioredoxin-dependent redox cycle upon addition of the second cysteine. Here we searched for examples of protein substrates where the number of redox-active cysteine residues has changed throughout evolution. We focused on cross-strand disulfides (CSDs), the most common type of forbidden disulfide. We searched for proteins where the CSD is present, absent and also found as a single cysteine in protein orthologs. Three different proteins were selected for detailed study—CD4, ERO1, and AKT. We created phylogenetic trees, examining when the CSD residues were mutated during protein evolution. We posit that the primordial cysteine is likely to be the cysteine of the CSD which undergoes nucleophilic attack by thioredoxin. Thus, a redox-active disulfide may be introduced into a protein structure by stepwise mutation of two residues in the native sequence to Cys. By extension, evolutionary acquisition of structural disulfides in proteins can potentially occur via transition through a redox-active disulfide state.

## Introduction

Thiol-based redox signaling is the collective name for biochemical pathways that regulate cellular processes by post-translational modification of sulfur moieties in cysteine (Cys) and methionine (Met) residues of proteins. These pathways are pathologically dysregulated in diseases of oxidative stress which include cancer, neurodegenerative diseases, heart disease and aging. A better understanding of these pathways is essential to diagnosis, treatment, and prevention of these diseases.

Cys residues are susceptible to a variety of modifications by reactive oxygen and nitrogen oxide species (ROS, RNS). Cys can be nitrosated, glutathionylated, and can form covalent bonds with other Cys. RNS such as nitric oxide (•NO) can mediate S-nitrosation to yield an S-nitrosothiol (RSNO). Other RNS, such as peroxynitrite (ONOO–), can also mediate S-nitration to yield S-nitrothiols (RSNO_2_). Sequential oxidation of Cys thiols yields sulfenic (–SOH), sulfinic (–SO_2_H), or sulfonic (–SO_3_H) acid derivatives. Reaction of protein thiols with low-molecular weight thiols such as glutathione (GSH) can yield mixed disulfides. Alternatively, oxidation by ROS or RNS can result in a disulfide bridge forming between two thiols, either within a protein chain or between protein chains (Wouters et al., 2011).

Reduction of these systems is effected in a variety of ways. For some disulfides, autoreduction within a protein may be assisted by mechanical stresses and the local electrostatic environment. For example, arsenate reductase (ArsC), an enzyme involved in detoxification of arsenic, transfers electrons through a series of Cys modifications, manifested largely as disulfide isomerizations, as part of its catalytic cycle (Messens et al., 2002). Two of the three disulfides formed in the oxidized states of ArsC are autoreduced, while the disulfide in the final oxidized state is reduced by thioredoxin (Trx) (Messens et al., 2002; Wouters et al., 2010). In general, reduction of surface accessible disulfides may be effected by the GSH redox buffer (Ostergaard et al., 2001), or specific thiol oxidoreductases such as Trx. GSH-Cys adducts may be reduced by glutaredoxins (Grxs), which are homologs to Trxs (Martin, 1995), and share similar binding modes of the protein substrate with Trxs.

It is now understood that a continuum of redox set points within proteins control cellular processes in response to a range of ROS fluxes. Low fluxes mediate homeostatic control of housekeeping redox processes, while higher fluxes mount stress and adaptive responses before a threshold is reached, triggering apoptosis, or programmed cell death. Partial loss of control of these processes is now believed to underlie aging (Humphries et al., 2006). Even higher levels of ROS can cause cellular necrosis.

In addition to its roles in housekeeping and the stress response, thiol-based redox regulation is also important in developmental processes in plants and animals. In plants, thiol-based redox regulation is involved in seed germination (Buchanan, 1980). In animals, the early embryo forms under reducing conditions (Hartley, 1960), but the embryonic environment becomes more oxidizing as the embryo grows (Hartley, 1960; Ibrahim et al., 2006). Redox-regulated processes govern the formation of substructures during embryo development by selective apoptosis.

The evolution of these systems is not well characterized. Among prokaryotes, the well-studied GSH system of eukaryotic redox regulation operates only in purple bacteria and cyanobacteria (Fahey, 2001; Mallick et al., 2002). In other prokaryotes, different redox buffers are utilized, for example acetyl-coenzyme A in gram positive bacteria (Hummel et al., 2005). It appears that the role of GSH as a redox buffer is predated by its role in detoxification. Glutathione S-transferases (GSTs) label xenobiotics with GSH, allowing them to be removed from the cell by specific transporters. GSTs are found in all classes of eukaryota and bacteria. Within GSTs, the GSH-conjugating site lies in a Trx-like domain (Atkinson et al., 2009). These data suggest that a common stress-related ancestor with a Trx-like fold may have pre-dated the evolution of the thiol-oxidase function now most commonly associated with the Trx-like fold.

In its role as a redox buffer, GSH is conjugated to reactive Cys of endogenous proteins, inducing conformational changes in the substrate proteins, and effecting a signaling cascade that evokes biological responses (Wouters et al., 2011). Conformational changes are generally small, involve the protein backbone, and are often accompanied by a local increase in protein disorder (Mallis et al., 2000; Wouters et al., 2011). Surface modification of proteins such as carbonic anhydrase by GSH results in significant disorder of the GSH distal to the covalent bond (Mallis et al., 2000). In this disordered state, GSH may act as a flipper, disrupting protein interactions. Thus, introduction of a single Cys into a protein may allow reversible GSH conjugation to occur, effectively introducing a simple redox switch into the protein.

If a second Cys is introduced into the protein sequence, the potential for disulfide formation exists. In favorable configurations and protein contexts, a bistable redox switch may be formed (Wouters et al., 2010). This has the potential to effect two different “active” states of the protein. For example, the protein may interact with certain protein partners with the switch in one redox state, and with other protein partners when the switch is in the alternate redox state.

**Figure 1:**
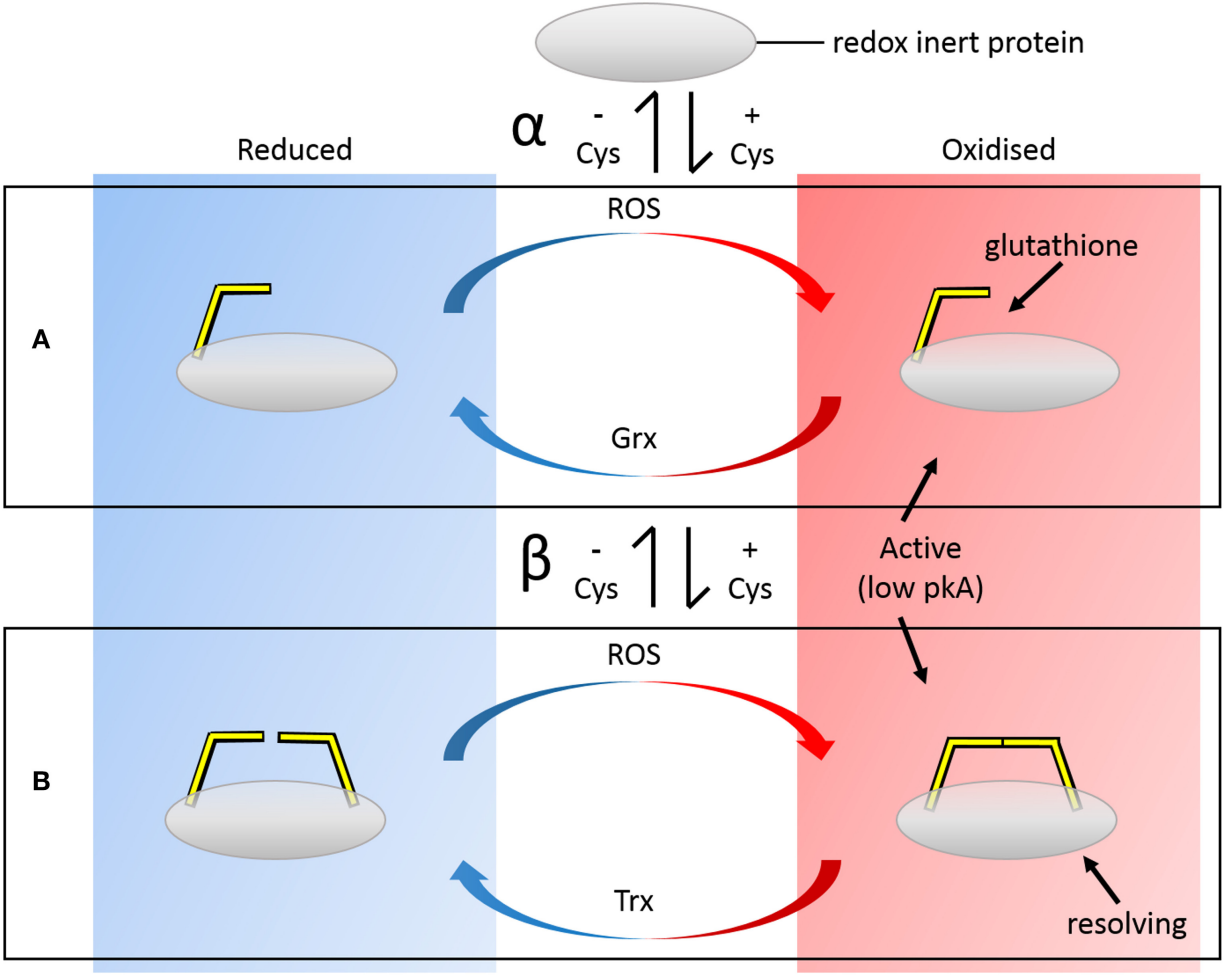
**Panels depict biochemical redox cycles of (A) single Cys and (B) disulfide-forming pair of Cys residues and their evolution (white central vertical panel)**. Incorporation of a single Cys (α) may make the protein immediately responsive to a range of oxidative modifications, including nitrosation and glutathionylation. Cys with lower pKas are more susceptible to modifications. Introduction of a second Cys (β) at a later stage may then enable disulfide formation, preventing overoxidation of the low pKA “active” Cys and effectively forming a bistable redox switch. Because Grxs are homologs to Trxs and share similar binding modes of the protein substrate, the complete redox cycle may potentially be immediately effected. These evolutionary modifications are reversible i.e., the resolving Cys may be lost, returning the protein to the Grx pathway etc.

How are these redox-regulated sites introduced into proteins? The introduction of potential disulfide-forming thiol pairs may be facilitated by the fact that both Cys do not need to be introduced into the protein chain simultaneously. This may seem obvious in isolation but, *in toto*, a number of constraints must be satisfied, and the likelihood of this occurring is greatly increased if the process can occur in a stepwise manner. Incorporation of a single Cys may make the protein immediately responsive to a range of oxidative modifications, including nitrosation and glutathionylation (Figure 1). In order for these modifications to be effective, the context of the Cys within the protein must be suitable. To be modifiable, the Cys must have a low pKa, a property that is largely determined by the electrostatic environment of the protein context. In addition, in order for GSH to be reductively removed from the Cys, Grx must be able to dock to the backbone near the modified Cys. Thus, there are additional requirements on the secondary structure and accessibility of the introduced Cys residue. Introduction of a second Cys at a later stage may then enable disulfide formation subject to further constraints. Because Grxs are homologs to Trxs and share similar binding modes of the protein substrate, the complete redox cycle may potentially be immediately effected. So the similarity in the constraints of 1-Cys and 2-Cys reduction facilitates this second Cys acquisition step, but an additional constraint is that the second Cys must be the resolving Cys and thus have a higher pKa than the existing Cys.

Anecdotally, a number of these 1-Cys/2-Cys homologs have been described (Wouters et al., 2010). An example where one protein is glutathionylated and a homolog forms disulfides is the Grx–Trx pair itself. Trx forms a disulfide between the Cys residues of the CXXC motif as part of its reaction cycle. Grx contains only the reactive N-terminal Cys of the CXXC motif which is glutathionylated during its reaction cycle. Another pair of families is the 1-Cys and 2-Cys peroxiredoxins (Prxs) (Copley et al., 2004). When the resolving Cys is present, Prxs are reduced by Trx. When it is not, they are reduced by Grx. In all the above examples, the additional Cys is the resolving Cys, suggesting the reactive Cys must come first in order for the mutation to become fixed in the population. These examples demonstrate evolutionary exaptation from the Grx pathway to the Trx pathway, or vice versa, does occur; and likely can be easily effected for other proteins.

The vast database of sequenced genomes of organisms is a valuable resource which can be mined for information regarding the evolution of these pathways, leading to a better understanding of their function and control. Here we searched for examples of protein substrates where the number of redox-active Cys residues has changed throughout evolution.

We focused on cross-strand disulfides (CSDs), a type of forbidden disulfide motif. Forbidden disulfides are a group of canonical disulfides that disobey elucidated rules of protein stereochemistry (Wouters et al., 2010). CSDs are metastable disulfides: their intermediate disulfide torsional energies render than more easily reduced than low energy structural disulfides, but they are more stable than high energy disulfides (Wouters et al., 2007, 2011). Their reduction is likely assisted by endogenous proteins. The postulated role of CSDs as canonical redox switches is supported by anecdotal information underpinning a functional redox role in specific examples (Haworth and Wouters, 2013). In particular, several CSDs are known Trx substrates (Maeda et al., 2005), and we have postulated recently that CSDs are, in general, cognate substrates of Trx (Haworth and Wouters, 2014).

Here, we focused specifically on protein orthologs where the CSD is present, absent and also found as a single Cys. We provide a number of examples and speculate on the evolutionary advantages of the changes.

## Materials and methods

We mined the Protein databank (PDB) (Berman et al., 2000) for proteins with CSDs, the most common type of forbidden disulfide. In a dataset of 29,261 disulfide-containing proteins, 195 unique protein clusters containing 235 CSDs were retrieved (Haworth and Wouters, 2013). These proteins were mapped to Uniprot (Magrane and Consortium, 2011) using the PDB identifiers. For each protein sequence in this CSD set, sequences in the corresponding 50% cluster of homologs from Uniprot were retrieved and aligned.

### Selection of proteins for study

The proteins used in this study were chosen from the above list based on two criteria—firstly, that there was good sequence evidence (i.e. more than one ortholog) that an intermediate 1-Cys state for the protein existed, and was retained in some species; and secondly, that there was evidence that the protein was redox regulated. From this set of CSD-containing proteins, three different proteins were selected for detailed study—CD4, ERO1, and AKT1. A fourth protein, DAPP1, satisfied the first criterion, but because we could find no evidence supporting its redox activity, it was not considered further. The corresponding identifiers for the selected proteins in Uniprot were P01730 (CD4_HUMAN), Q96HE7 (ERO1A_HUMAN), and P31749 (AKT1_HUMAN). Data for the corresponding protein structures 4h8w, 3ahq, and 1unr was retrieved from PDBsum (De Beer et al., 2014).

### Multiple sequence alignment and tree generation

For each seed sequence, an initial alignment was performed in Uniprot using the sequences in the 50% sequence identity cluster (Clusterref_50) with the “Align” option. The raw multiple sequence alignment from Uniprot was loaded into Jalview (2.8.1) (Waterhouse et al., 2009) for editing, visualization and analysis. Masking was performed to remove ambiguous regions of the alignment, and the initial tree was generated with the Neighbor-joining method using the BLOSUM 62 substitution matrix. Additional sequences were added to the alignment using a combination of Blast searches (Altschul et al., 1990) of Uniprot, and joining of additional 50% sequence identity (ID) clusters to provide further evolutionary depth when necessary. The generated tree in Newick format was loaded into the evolutionary tree builder MEGA6 (Tamura et al., 2013) and the root chosen using an outgroup from the accepted species tree (Murphy and Eizirik, 2009). The figure for the final tree was created in MEGA6 and further manipulated in Illustrator 6 (Adobe Systems). The number of Cys residues in the CSD motif was mapped onto the phylograms manually.

#### CD4

For CD4, the human cluster Uniref50_P01730 containing 42 sequences from 20 different species was initially retrieved. Multiple sequences were excluded: including 15 partial sequences, seven Refseq sequences, an uncharacterized protein, a testis cDNA sequence and two surface antigen sequences; resulting in an initial alignment built on 16 sequences from seven different species. Since the sequences in the cluster did not provide enough evolutionary information, we extended our analysis using a Blast search (Altschul et al., 1990) in Uniprot using P01730 as a template. The results from the Blast search were again filtered, excluding alternate isoforms from the same species, resulting in an additional 125 CD4-like sequences being added to the alignment. Masking was performed to remove alignment ambiguities near gaps. As the alignment was ambiguous in the region of the CSD in fishes, turtle, duck, snakes, and frogs, these sequences were excluded from further consideration. The final tree was built on an alignment of 51 sequences from 50 different species using residues homologous to 85–129, 150–230, 233, 236–244, 281–302, 307–348, and 351–373 of the human sequence. The disulfide of interest forms between Cys residues at positions 154 and 184 of the human sequence P01730 (130 and 159 in the PDB structure: 4h8w). The complete phylogram is shown in Supplementary Figure 1. Further analysis of sequence conservation was performed with Jalview on sequence subsets. In one subset, the sequences of 16 primate and three muroid sequences, where the disulfide was conserved, were analyzed separately in the vicinity of the CSD. In two additional subsets, separate pairwise alignments of human and galago CD4, and mouse and Chinese hamster CD4 were performed as a part of more detailed analyses (Supplementary Figure 2).

#### ERO1

For ERO1, the cluster Uniref50_Q96HE7 built on the human sequence, containing 66 protein sequences from 37 species, was used in the initial alignment. Most of the sequences were ERO1A and ERO1B genes in higher animals. In order to retrieve more evolutionary information, a Blast search was performed in Uniprot using the human ERO1A protein Q96HE7 as the template. This retrieved ERO1-like sequences from divergent species including worms, beetles, lancelet, fishes, fruit flies, and mosquitoes. The 50% identity cluster Uniref50_L7MC09 built on the *Rhipicephalus pulchellus* sequence, with sequences from lancelet (C3Z2H2), red flour beetle and polychaete worms, was incorporated, and an additional Blast search using the ERO1 protein of *Capitella teleta* (Polychaete worm) was performed. The resulting alignment of 177 sequences was further refined by choosing a representative sequence for branches with very small branch lengths. Masking was performed and a tree built from the final alignment containing 119 sequences using regions 33–58, 61–103, 108–115, 137–144, 157–160, 174–210, 239–270, 275–287, 295–324, 328–335, 338–363, 371–386, 390–393, 402–418, 433–438, and 444–456 homologous to the human ERO1. A Newick tree was generated in Jalview and loaded into MEGA6, where the Black flying fox sequence (Uniprot id: L5K102) was chosen as the root. The two CSDs of interest are nested in the sequence at positions 35 and 48 and 37 and 46 (PDB: 3ahq and Uniprot: Q96HE7). A complete phylogram is shown in Supplementary Figure 3.

#### AKT

For AKT, the Uniref50_P31751 cluster was retrieved with 121 sequences from 69 organisms. Of these, 54 sequences from 38 different organisms, from human to snake, were mapped to Uniprot and the initial alignment was built. This was merged with UniRef50_Q17941, a 50% sequence ID cluster built on *Caenorhabditis elegans* AKT1 (Q17491) which mapped to 19 Uniprot sequences from 18 different organisms, mostly arthropods; and the 50% cluster Uniref50_Q9XTG7 built on *C. elegans* AKT2 with nine mapped sequences from sea squirt, *C. vulgaris* and platyfish. A Blast search was performed with the Honeybee AKT1 (H9KF44) as the template. After removing the duplicates, the resulting sequences were aligned in Uniprot and merged with the previous alignment. After masking, the final alignment was built with 262 sequences with residues 28–45, 48–71, 76–113, 146–210, 215, 230–266, 269–302, and 335–427 homologous to the human AKT1 protein P31749. A Newick tree was generated and loaded into MEGA6. The land crab sequence was chosen as the root. The disulfide of interest is formed between Cys 60 and Cys 77 of the human AKT1 protein (PDB: 1unr and Uniprot: P31749). The complete phylogram is shown in Supplementary Figure 4.

### Physicochemical properties of CSDs

To further investigate the lability of the CSDs of interest we calculated the torsional energies of the disulfide bonds; and, where possible, the pKas of the two involved Cys. Torsional energy calculations were performed using an online torsional energy calculator^1^ based on input dihedrals, which uses a combined quantum chemical (**χ**_2_, **χ**_3_, **χ**′_2_) and empirical calculation (**χ**_1_, **χ**_1_′) described in detail elsewhere (Haworth et al., 2010). Dihedral angles were calculated using Pymol^2^. Calculations of pKa for individual Cys residues were performed with Propka^3^ which gives an approximation of the pKa based on a solution of the Boltzmann equation (Rostkowski et al., 2011). The only reduced structure available was for AKT (PDB: 1unq).

## Results

We previously mined the PDB for proteins with CSDs, the most common type of forbidden disulfide (Haworth and Wouters, 2014). Forbidden disulfides are strained disulfides that occupy recognizable contexts in primary and secondary structure of proteins. The contexts were originally declared “forbidden” by analyses that considered the geometric constraints of disulfide formation and hydrogen bond formation in secondary structure (Richardson, 1981; Thornton, 1981). Later work has shown that disulfides in these strained contexts do occur, despite their strain, which likely relates to their functional redox role in proteins (Wouters et al., 2010; Haworth and Wouters, 2013). The frequency of CSDs in solved protein structures is increasing, likely due to better control of redox conditions during structure solution (Wouters et al., 2010). Because CSDs can act as redox switches, failure to control conditions carefully results in the proteins adopting different structural states. This heterogeneity adversely affects the crystallization process.

CSDs have been introduced into protein sequences extensively throughout evolution, especially in molecules of the immune system, particularly the adaptive immune system (Wouters et al., 2007). In order to study this process in the selected proteins of interest, we scanned homologs of proteins with CSDs for sets where the disulfide was not completely conserved. Three proteins were chosen where a CSD has been introduced relatively recently in evolutionary history; and where the 1-Cys evolutionary intermediate state was preserved in some species: CD4, ERO1, and AKT. For each of these proteins, alignments of the homologs were created from Uniprot clusters and Blast searches. Alignments were masked and evolutionary trees created as described in detail in the methods.

### CD4

CD4 is a critical protein of the adaptive immune system, which may be expressed on the surface of some lymphocytes, including T helper cells, monocytes, macrophages, and dendritic cells. CD4 T helper cells signal to other immune cells, such as CD8 killer cells, to alert them to the presence of infectious agents which must be targeted. CD4 is a co-receptor for major histocompatibility complex (MHC) class II molecules, and the primary cell surface receptor for Simian immunodeficiency virus (SIV)/Human immunodeficiency virus (HIV) (Dalgleish et al., 1984).

In CD4+ T cell antigen receptor-mediated signaling, the polymorphic regions of the MHC class II proteins engage the receptor complex, whereas the non-polymorphic regions engage the CD4 co-receptor (Parnes, 1989). The TCR/MHCII/CD4 super-complex then recruits intracellular molecules such as LCK to effect signaling.

**Figure 2:**
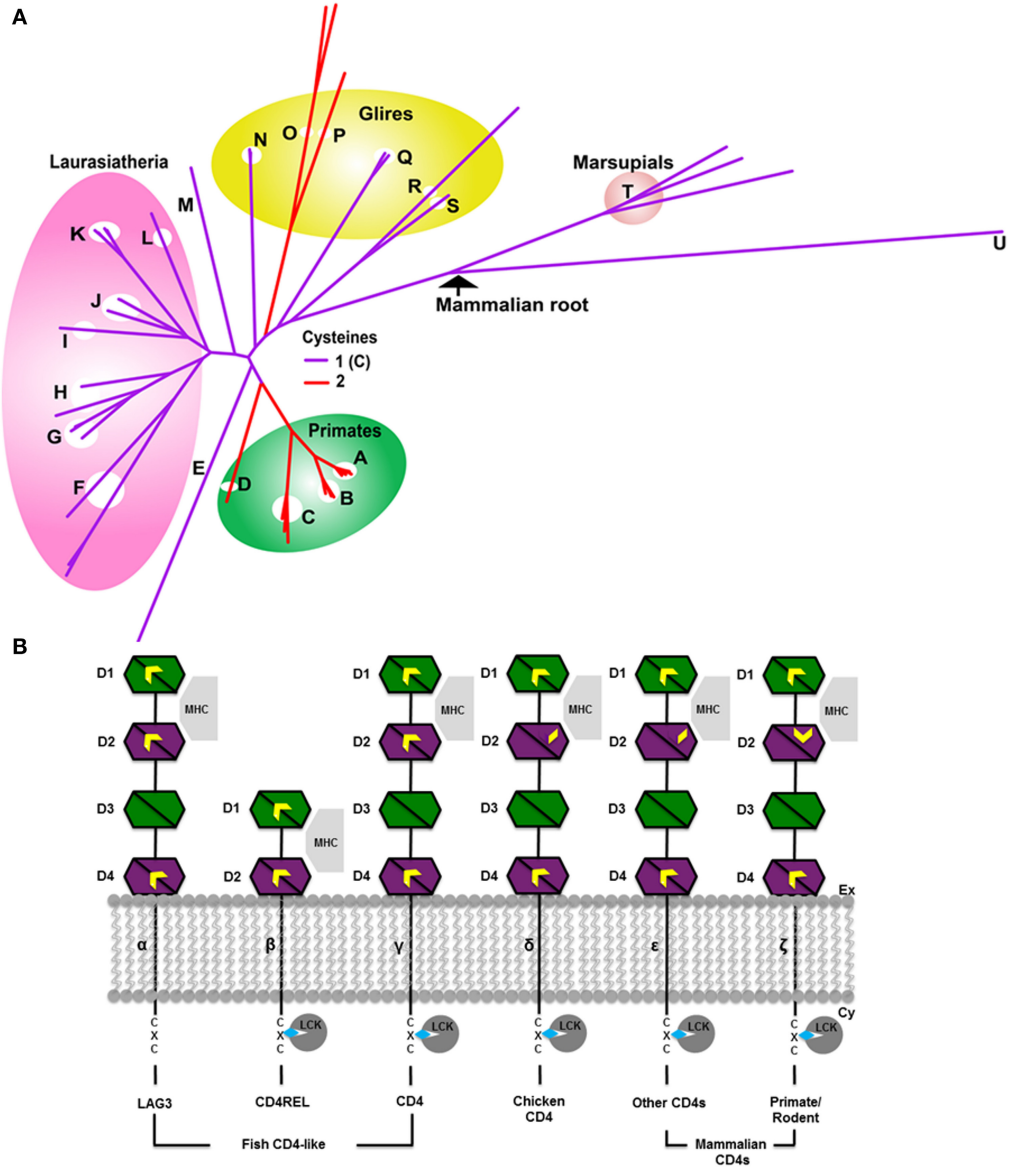
**Evolution of CD4 (A) phylogenetic tree in mammals**. The color of the branches indicates the number of Cys of the CSD motif in D2. The CSD is found in species with red branches. Species with purple branches have only the C-terminal Cys. The CSD was likely acquired in primates (A–D) around 75 MYA, and independently in muroid rodents *viz* mice, rats (O) and hamsters (P), after the divergence of squirrels (Q, sciuridae), also around 75 MYA. Acquisition of the CSD in muroids was accompanied by rapid evolution of the CD4 protein as evidenced by the long branch lengths of this clade (upper red branches). The four major clades of Eutherian mammals are Afrotheria (including elephants E); Xenartha (none depicted); Laurasiatheria (F–L, shown in pink) and Euarchontaglires (primates, A–D, green circle; glires, N–S yellow circle; and treeshrews, M) (Murphy et al., 2001) Key: A, Lesser apes; B, Greater apes; C, Marmoset (Platyrhines); D, Galago; E, African Elephant; F, Bats; G, Panda/Mink/Ferret; H, Dog and cat; I, Pig; J, Dolphin, Whale and Camel; K, Sheep, Bovine, Goat; L, Horse; M, Tree shrew; N, Rabbit; O, Mouse and rat; P, Chinese hamster; Q, Squirrel/wood chuck; R, Guinea pig; S, Mole rat; T, Wallaby and opossum; U, Platypus. **(B)** Evolution of CD4 and related molecules showing important thiol-containing sites. Molecules are depicted from the more ancestral forms of the molecule (leftmost) to more recently evolved forms to the right. The CD4 homologs consist of two to four extracellular Ig domains, a single transmembrane region and a short cytoplasmic tail. Three important thiol-bearing functional sites have been identified: the CXC motif in the cytoplasmic tail, the between-sheet disulfide in D1, and the cross-strand disulfide (CSD) in D2. The CXC motif is present in all CD4 orthologs **(β–ε)**, but not in the CD4-like LAG3 molecule. The between-sheet disulfide in D1 is present in CD4 and LAG3 **(α–ζ)**. In some teleosts, such as trout and fugu, the Cys on strand B is lost, leaving a single unpaired Cys on strand F. D1 can be definitively identified in CD4REL because it is distinctively encoded on two exons in molecules **β–ε** (Laing et al., 2006). It is involved in interaction with MHC II molecules. Thiols in the D2 site have evolved over time. Originally D2 contained Cys residues on strands B and F, typical of Ig domains (**α–γ**). Around 250 MYA, the thiol on strand B was lost (**δ–ε**). Around 75 MYA, a new thiol appeared on strand C allowing the novel CSD to form upon oxidation (**ζ**). **α–γ** are CD4-like proteins which evolved in a trout ancestor around 450 MYA. Interacting proteins are shown in gray: all proteins bind MHC class II; the CD4-related molecule LAG3 (**α**) binds MHC but not LCK. D3 and D4 contain glycosylation sites which may serve to fend off adventitious interactions with other membrane proteins (Laing et al., 2006). Ig domains are depicted as bisected hexagons with each half representing one sheet: V-like domains are depicted in green; C-like domains in magenta. A CXXC motif in LCK binds to the CXC motif of CD4 via Zn^2+^ (depicted as a blue diamond) (Lin et al., 1998). The extracellular and cytoplasmic sides of the membrane are labeled on the right.

The CD4 molecule is a single-pass transmembrane protein with 4 extracellular immunoglobulin (Ig) domains and a short cytoplasmic tail. The Ig domains comprise a duplication of a pair of Ig variable (IgV) and Ig constant (IgC) domains arranged in tandem (Figure 2B). Structurally, IgV domains are slightly larger than IgC domains, having more strand elaborations on the basic Ig domain fold (Halaby et al., 1999). Ig domains are extremely common domains that are frequently involved in redox regulation (Wouters et al., 2010; Haworth and Wouters, 2013). Ig domains and their antecedents, the fibronectin type III (FIII) domains are found in many proteins of both the innate and adaptive immune system including cytokine receptors (FIII domains); and Ig domain-containing MHC homologs such as MICA and RAE-Iβ (Wouters et al., 2007). Ig domains consist of two β sheets that form a sandwich, typically containing seven to nine strands. The seven-stranded variety is labeled from A to G. Additional strands in larger Ig domains (such as the variable domains) are typically found between the C and D strands and are labeled C′, C″ etc. In general, Ig domains are distinguished from FIII domains by an intersheet disulfide that links strand B on one sheet to strand F on the other.

Three regions of CD4 have been implicated to date in important protein-protein interactions (Figure 2B). The region of human CD4 that interacts with MHC class II lies mainly within the C″ and D strands of domain 1 (D1), the N-terminal IgV domain (Wang et al., 2001). A second region, located in the short cytoplasmic tail, is a CXC motif that mediates the interaction with lymphoid cell kinase (LCK or p56LCK) necessary for T cell activation (Laing et al., 2006). The third region, which contains the CSD of CD4, is located in D2, the N-terminal IgC domain, joining two β-hairpins. The CSD is formed by a disulfide bond between Cys 130 and Cys 159 in the structure (PDB: 4h8w).

The CSD has been demonstrated to be reduced during the process of T-cell signaling, and its reduction is also co-opted during entry of the HIV viral envelope glycoprotein (gp120) into the cell. During T-cell signaling, it must be reduced in order for the TCR/MHCII/CD4 receptor complex to form: a process that requires CD4 dimerization and domain swapping (Cerutti et al., 2014). Reduction of the CD4 CSD is mediated by Trx, Grx1, and PDI *in vitro* (Gallina et al., 2002; Matthias et al., 2002; Auwerx et al., 2009). Further investigation of the role of the CSD of CD4 in T-cell signaling has recently been performed using four Cys mutants: C159A, a mutant of the putative active Cys of the CSD; C16A/C84A, a double mutant deleting the disulfide from D1; C130A/C159A, a CSD deletant; and C16A/C84A/C130A/C159A, a D1 disulfide and CSD deletant (Cerutti et al., 2014). Experiments with these mutants demonstrated that CD4 binds gp120 of HIV as a reduced monomer; and that CD4 requires reduction of either the D1 or the D2 disulfide to bind gp120, but not both (Cerutti et al., 2014). A redox potential of -241 mV has been determined for the CSD by titration against DTT (Matthias et al., 2010). The higher efficiency of Trx reduction of the CSD suggests the reduction is likely performed by Trx *in vivo* (Cerutti et al., 2014).

**Figure 3:**
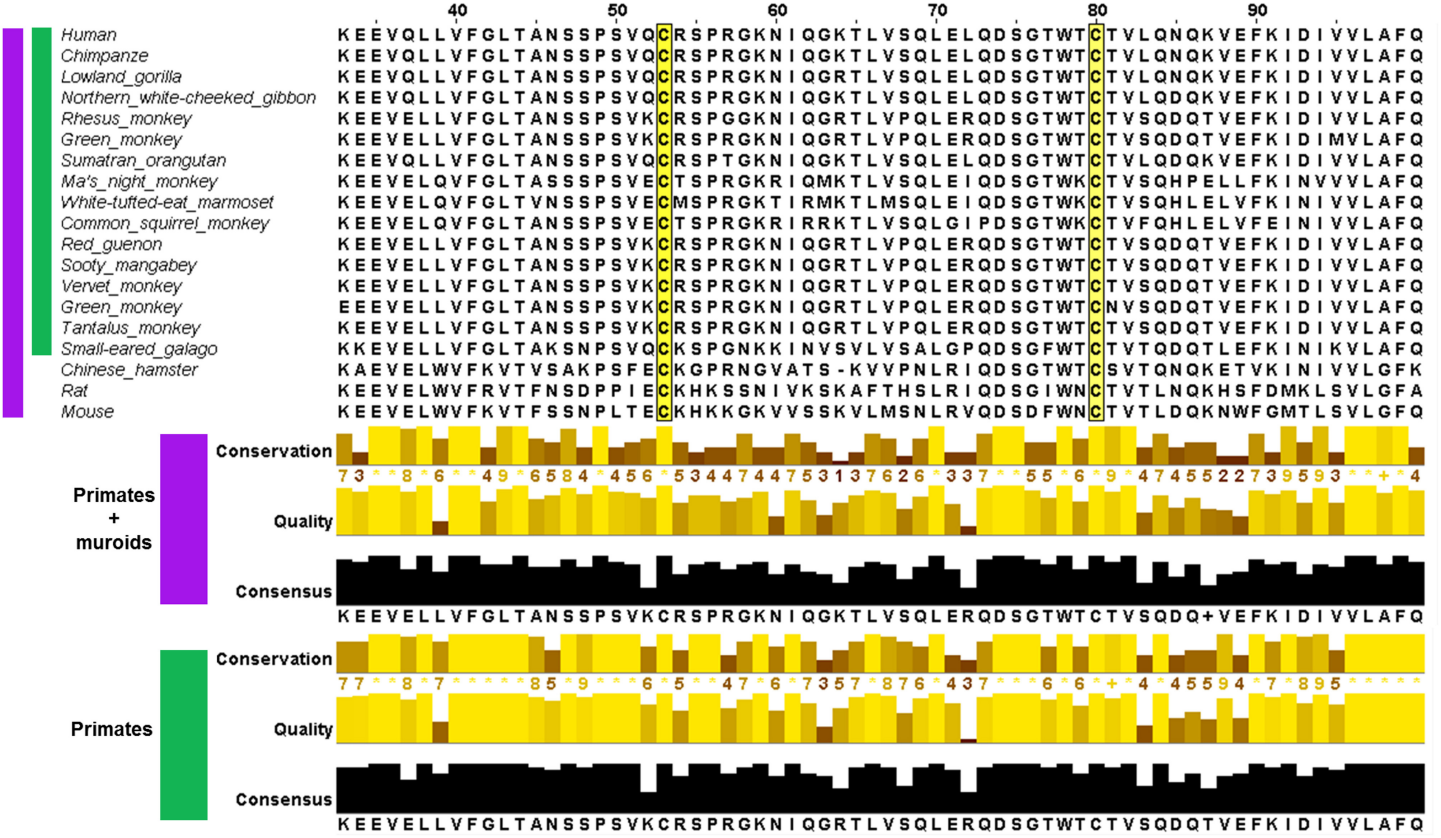
**Multiple sequence alignment of CD4 in the region of the CSD in species where both Cys are conserved (upper panel) and measures of its conservation (lower panel) across two groups: primates and muroids (purple), and primates only (green)**. For each group, the conservation of the amino acid residue in each column, quality (BLOSUM62 score based on observed substitutions) and consensus (most common residue and its proportion for each column) as determined by Jalview (Waterhouse et al., 2009) is shown. The sequence alignment of CD4 showed less conservation in the vicinity of the CSD motif. In the portion depicted, the average conservation across the primate + muroid lineages is 6.2 for residues 53–80 (CSD region highlighted in yellow) and 7.9 within the primate group.

The phylogenetic tree for CD4, built from 51 sequences from 50 species is shown in Figure 2A. CD4 is thought to have arisen relatively recently in teleost fish following duplication of a two Ig domain-containing ancestral gene, CD4REC (Laing et al., 2006). In trout, CD4, CD4REC and the related protein lymphocyte-activation gene 3 (LAG3) share many similar features (Figure 2B) (Laing et al., 2006). The CSD is not present in D2 of trout CD4 which instead contains a disulfide between strands B and F (Laing et al., 2006), typical of most Ig domains (Halaby et al., 1999). The full alignment shows that between the divergence of bony fish and chicken, the Cys on strand B was lost by mutation. The homologous residue is a leucine (Leu) (143 in P01730 and 118 in PDB: 4h8w) in human CD4, and a single unpaired Cys residue remains on strand F in D2 of the chicken sequence. It seems likely this single Cys is a glutathionylation site in chicken. Based on the phylogenetic tree, the acquisition of the second Cys on strand C enabling CSD formation likely occurred independently in two different clades, both within euarchontaglires, one of four large clades of placental mammals (Murphy and Eizirik, 2009). The tree in Figure 2A shows only mammals. The CSD is found in primates, which emerged around 75 million years ago (MYA); and was acquired independently in muroid rodents (mice, rats, and hamsters), after the divergence of squirrels (sciuridae), also around 75 MYA (Murphy and Eizirik, 2009). Acquisition of the CSD in both clades was accompanied by rapid evolution of the sequence between the two Cys residues in D2, as shown in the partial alignment in Figure 3 and in the full pairwise sequence alignments in the supplementary data (Figures S2b, S2c). The pairwise identity for the entire CD4 molecule between human and galago is 74%, and between rat and chinese hamster is 66%; but the pairwise sequence identity over a 52-residue window surrounding the CSD is 63% for the pairwise primate sequences, and 44% for the pairwise muroid sequences. This demonstrates that rapid remodeling of the sequence surrounding the CSD has occurred during the last 75 MYA in both the primate and muroid clades. See further notes on this event in the Discussion.

### ERO1A (endoplasmic reticulum oxidase)

ERO1 is an essential oxidoreductase involved in disulfide formation of nascent proteins in the endoplasmic reticulum (ER). It acts by reoxidising protein disulfide isomerase (PDI), the enzyme catalyzing disulfide formation. It has been extensively studied in yeast (Frand and Kaiser, 1998). The protein contains multiple redox-active disulfide bonds. In human ERO1, two disulfide-forming Cys pairs, Cys 94/Cys 99 and Cys 394/Cys 397, constitute the redox-active enzymatic center. The first pair of thiols are regulated via isomerisation with a second pair of redox-active thiols to form a pair of nested disulfides between non-consecutive Cys in the sequence (Cys 99–Cys 104, Cys 94–Cys 131) (Hansen et al., 2012).

**Figure 4:**
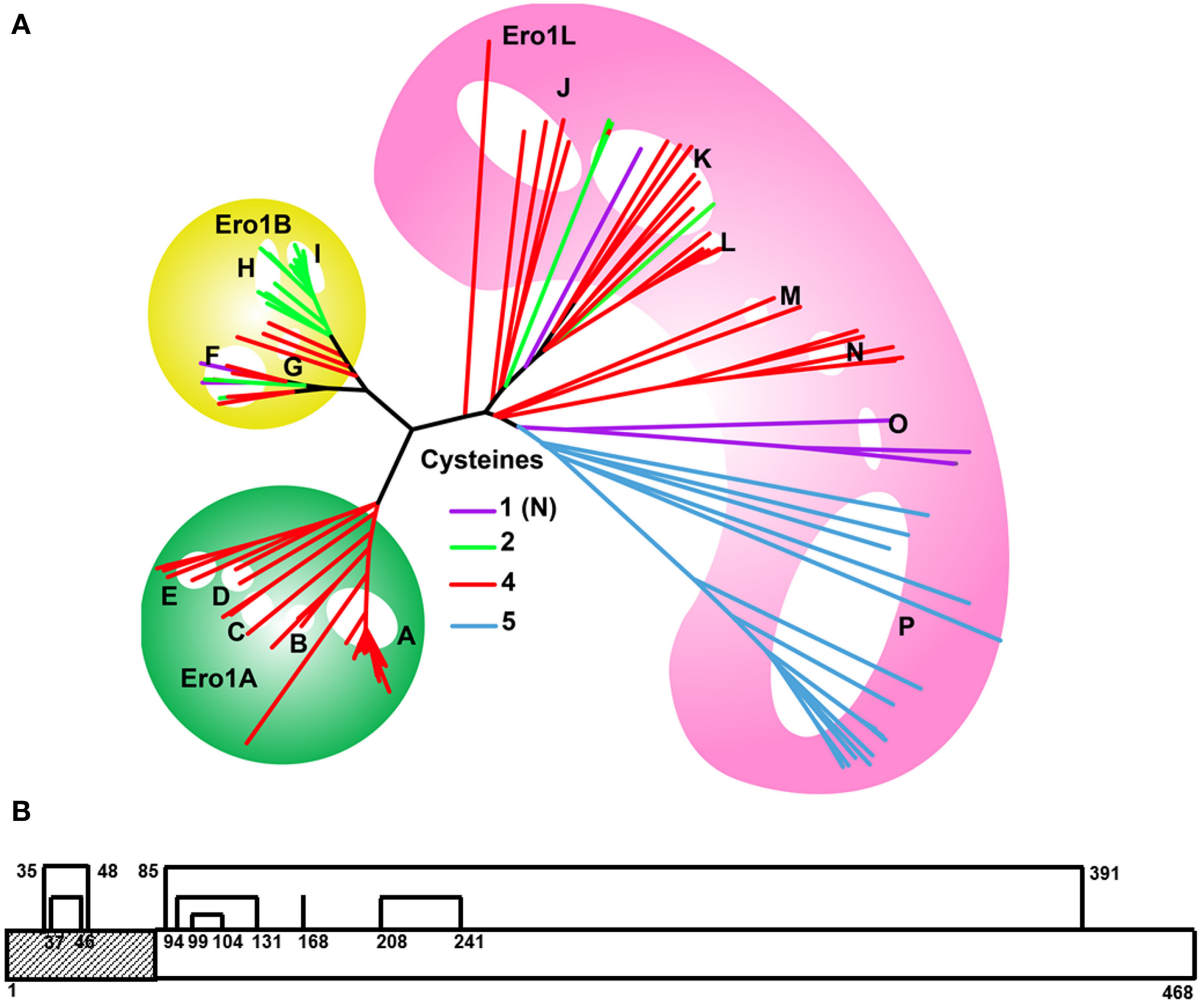
**(A)** Evolution of the ERO1 gene family. Branches are colored according to the number of Cys present in the NTR of each species, as shown in the legend. A single ERO1-like ancestral gene (pink background) is present in eukaryotes from yeast to lamprey (J–P). With the exception of plants (P), the majority of these genes have four Cys in the NTR (red branches). ERO1 duplicated prior to the divergence of bony fishes. ERO1A (green background) genes retained the nested Cys motif, as does ERO1B (yellow background) in early diverging species such as fish, sharks, and amphibians (F, G). However, later diverging species such as birds, reptiles, and mammals (H, I) have only two Cys, homologs to the inner pair of CSD-forming Cys. Plants (P) have an additional Cys, C-terminal to the nested Cys motif. Selective loss of Cys residues has resulted in a single N-terminal Cys in some ERO1 genes (purple branches) such as in ERO1L in tapeworm (O) and Silk moth (K), and ERO1B in the Japanese ricefish. Key to branches: A, Mammals; B, Birds; C, Frog and turtle; D, Shark and Spotted gar; E, Fish; F, Fish; G, Shark, Frog and Coelacanth; H, Birds/Snake/Chameleon; I, Mammals; J, Sea squirt/Oyster/Sea urchin; K, Flies (Mosquito/Beetle/Bugs); L, Ants; M, Lancelet and Polychaete worm; N, Worms; O, Tapeworms; P, Plants. **(B)** Line diagram of ERO1 showing the disulfides and NTR. The two CSDs are the two nested disulfides in the NTR region. The NTR region comprising residues 1–55 is typically deleted in yeast functional assays. An equivalent region is typically deleted in ERO1α (Hansen et al., 2012). Interestingly, this region is not generally deleted in ERO1B (Pagani et al., 2000).

Near the N-terminus of the sequence, two additional disulfides which form a nested pair, are the CSDs present in the structure of human ERO1 (Figure 4B). In the human sequence, the two CSDs are formed between Cys 35 & Cys 48 and Cys 37 & Cys 46. These disulfides are deemed to have a “structural role” because they do not appear to be involved in thiol-disulfide exchange with the enzymatic center (Inaba et al., 2010), nor are they conserved across all ERO1 genes. However, the identity of these two disulfides as CSDs suggests a redox role, possibly as substrates of a Trx-like enzyme (Haworth and Wouters, 2013). The role of the Cys residues of the N-terminal thiol region (NTR) has not been investigated by mutation but constructs of human ERO1A and yeast ERO1 lacking the NTR are often used for functional assays (Cabibbo et al., 2000; Sevier et al., 2007; Hansen et al., 2012). This region may have a regulatory role.

The phylogenetic tree for ERO1, built from 119 sequences is shown in Figure 4A. Originally present as a single gene, ERO1 duplicated prior to the divergence of bony fishes. The single gene in organisms predating bony fishes is generally termed ERO1-like (ERO1L) because it is clearly homologous to yeast ERO1 but has diverged in some crucial aspects, for example the yeast sequence has a long C-terminal tail. Yeast ERO1 has diverged sufficiently from human ERO1 that a meaningful sequence alignment over the full mask region employed for the phylogenetic tree is not possible. Of the duplicated daughters, ERO1A is ubiquitously expressed. This gene is located on chromosome 14 in humans. The other daughter, ERO1B, expressed in secretory tissues such as the pancreas, is found on chromosome 1 in humans. In metazoan ERO1L and ERO1A, the NTR has four Cys residues homologous to the CSD residues of human ERO1A, suggesting the CSDs are conserved across the group. In ERO1B, the four Cys are retained in the NTR of fishes and sharks, but only two Cys, homologous to those forming the inner CSD, are present in ERO1B of amphibians and higher teleosts. The presence of four Cys or two Cys in the NTR of ERO1B homologs partitions with sea and land animals respectively. Similar to ERO1B, in yeast ERO1, only the inner Cys pair is present.

What is the significance of Cys mutations in the NTR to the function of ERO1 homologs? As proteins rich in disulfides are highly expressed in secretory organs, the cells in these tissues are more susceptible to ER stress. Thus, ERO1B is likely to be under increased demand for disulfide formation in the secretory tissues where it is expressed compared to ERO1 homologs in other tissues. In addition, the emergence of animals onto land likely increased the importance of secretory organs in dealing with the effects of dehydration. The fact that one of the CSDs is lost in the NTR of ERO1B found in secretory tissues of land animals, suggests its loss is associated with increased activity of ERO1B. This suggests the NTR may have a role in inhibition of ERO1. The loss of the outer CSD of ERO1B in land animals may have resulted in decreased inhibition, allowing these organisms to cope with the greater demands for disulfide bond formation in their secretory organs. The fact that NTR-deletants are typically used for functional assays of ERO1A and yeast ERO1 is consistent with this hypothesis.

### AKT

Kinases of the AKT family are cytoplasmic proteins forming essential components in growth factor signaling pathways, activated downstream of the membrane-bound phospho-inositol-3 kinase (PI3K). The AKT genes are serine/threonine-protein kinases involved in regulating many processes such as metabolism, proliferation, cell survival and growth, and angiogenesis. AKT1 (RAC-α) is encoded on chromosome 14 of the human genome. AKT2 (RAC-β), which acts in the insulin signaling pathway, is encoded on chromosome 19. Both AKT1 and AKT2 are activated by platelet-derived growth factor. AKT3 is expressed in the brain.

The protein consists of three domains: an N-terminal Pleckstrin homology (PH) domain, a protein kinase domain, and a C-terminal AGC kinase domain. The PH domain is involved in membrane targeting of AKT via binding of phospholipids. The CSD is formed between Cys 60 and Cys 77 in the PH domain (PDB: 1unr). Crystallographic evidence supports redox activity of the disulfide (Fan et al., 2009). The reduced structure is PDB 1unq. It undergoes a morphing transition: a plastic deformation involving a large-scale conformational rearrangement of the polypeptide backbone which is likely regulated by redox activity of the CSD (Fan et al., 2009). Cys 77 can be modified by methylglyoxal to form an advanced glycation end product (AGE). Methylglyoxal is a byproduct of several metabolic pathways including threonine metabolism, lipid peroxidation and glycolysis.

Like CD4, AKT has multiple redox-regulated thiol sites. In AKT2 a redox-regulated order/disorder transition, modulated by reduction of the Cys 297/Cys 311 disulfide in the T loop, controls exposure of a key phosphorylation site at Thr 309 (Huang et al., 2003; Fan et al., 2009). Formation of the T loop disulfide is proposed to inhibit kinase activity by recruiting the cognate phosphatase (Leslie, 2006). Grx reduces AKT, although it is not clear whether it acts at the CSD, the T loop disulfide, or some other site (Murata et al., 2003). Modification of Cys 77 by methylglyoxal results in activation of AKT1, promoting proliferation of vascular smooth muscles (Chang et al., 2011). Overexpression of an AKT1 Cys77Ser mutant increased cell proliferation and DNA synthesis which could not be augmented by methylglyoxal treatment like the wild-type.

**Figure 5:**
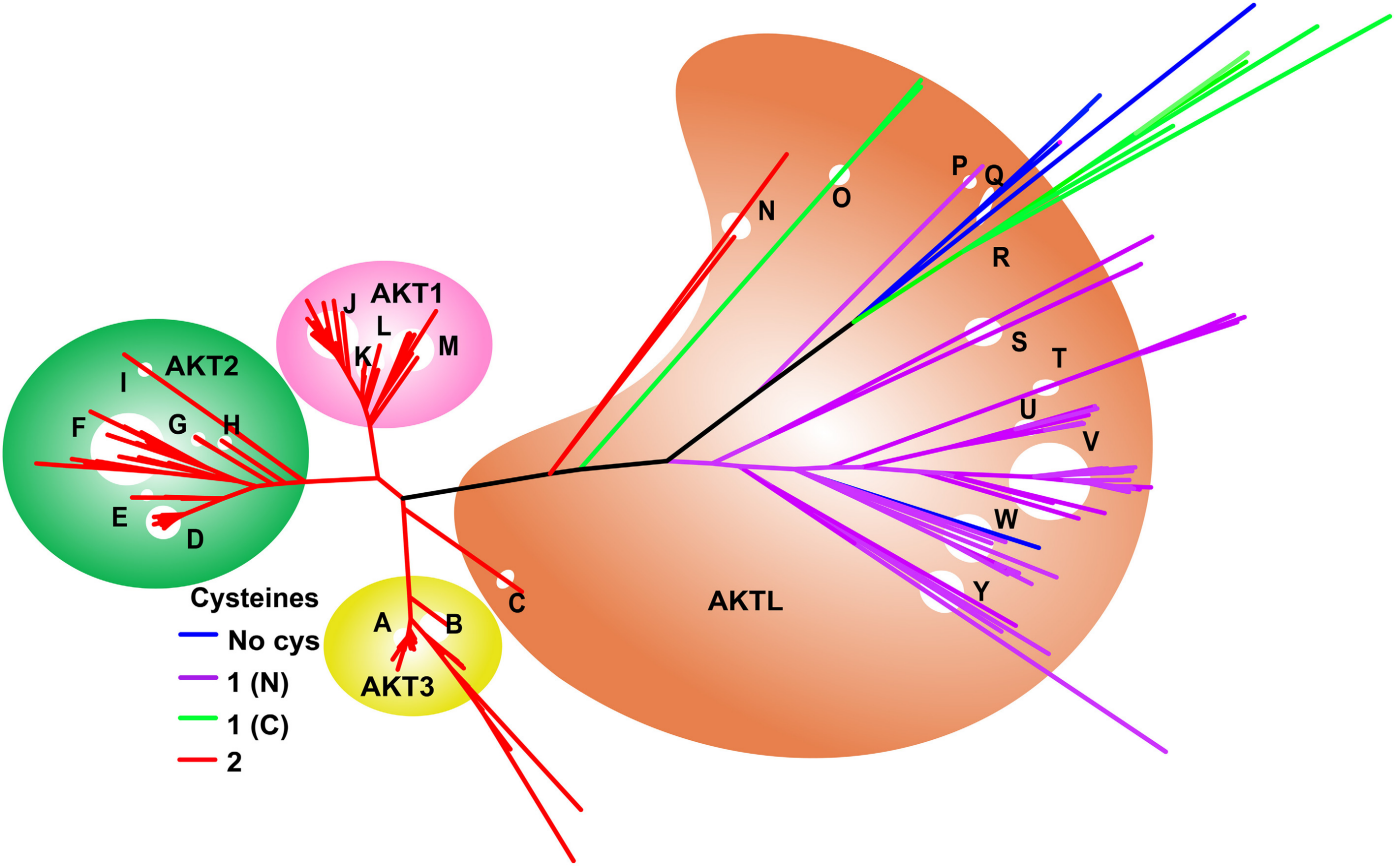
**Evolution of AKT genes showing the presence of the CSD**. Colors of branches denote the number of Cys residues homologous to those of the CSD present in each species. Reversion to a single C-terminal Cys in the Chinese tree shrew is accompanied by rapid diversion of the sequence from other mammalian sequences. Keys to branches A, Mammals, birds, reptiles; B, Fishes; C, Sea lamprey; D, Mammals; E, Snakes; F, Spotted gar, salmon, and fishes; G, Frogs; H, Elephant fish; I, Zebra fish; J, Mammals; K, Tasmanian devil, Opposum, Turtle, and Birds; L, Frog and Coelacanth; M, Fishes; N, Acorn worm; O, Sea squirt; P, Trichina worm; Q, Eye worm, Pig round worm, and Pinewood nematode worm; R-*C. elegans*, pole worm and nematode worm; S-Y-Arthropods including Lepidoptera (T) and Diptera (U,V).

The phylogenetic tree for AKT built from 262 sequences is shown in Figure 5. The AKT gene has triplicated from the original ancestor: The original AKT-like (AKTL) gene is found in metazoa ranging from *C. elegans* to lamprey. An initial duplication, occurring after the divergence of lamprey, a jawless fish, was followed shortly after by a second duplication. AKTL genes predominantly have a single N-terminal Cys, for example all arthropods; but orthologs exist with no Cys (clade Q—eye worm, pig round worm and pinewood nematode worm; and the red flour beetle within clade W, Coleoptera); and a single C-terminal Cys (clade R-*C. elegans*, pole worm and nematode worm, clade O—Sea squirt). Clade N is the earliest metazoan with both Cys of the CSD. This group includes the hemichordate acorn worm and a placazoan, a basal form of invertebrate. Lamprey is the highest organism containing a single AKT gene with both Cys of the CSD. Subsequent to the divergence of lamprey the AKT gene underwent a triplication, with all daughters: AKT1, AKT2, and AKT3, retaining the CSD.

### Physicochemical properties of the CSDs

The physicochemical properties of the CSDs of interest to this study are listed in Table 1, along with those of three control disulfides. Two positive controls are barley α-amylase/subtilisin inhibitor (BASI) Cys 144–Cys 148, a CSD which is a preferred substrate of Trxh; and DsbD Cys 103–Cys 109, a CSD which is the active site disulfide in the N-terminal domain of DsbD, which is reduced *in vivo* by the Trx-like C-terminal domain of DsbD. A negative control is BASI Cys 43–Cys 90 which is reduced by Trxh, but is not a preferred substrate (Maeda et al., 2006). All the CSDs adopt the right-handed staple conformation and have medium torsional energies (10–17.5 kJ.mol^−1^) typical of CSDs, except the outer disulfide of the ERO1 nested pair. This disulfide between Cys 35 and Cys 48 adopts a much higher energy *cis* GGS′ conformation (Haworth et al., 2007). Its extremely high torsional energy is consistent with an auto-reduction process; the Cys of this disulfide could potentially isomerize with Cys 37 and Cys 46 or may even be mechanically reduced. The negative control in BASI between Cys 43 and Cys 90 is a low energy disulfide in a right-handed spiral conformation, typical of structural disulfides (Haworth et al., 2007). In summary, with the exception of the Cys 35–Cys 48 CSD of ERO1, all the CSDs in the proteins of interest had conformations and torsional energies consistent with CSDs which are preferred substrates of Trx-like enzymes.

**Table 1:** **Dihedral angles calculated for the disulfides of interest in the proteins studied with the torsional energies and their respective conformations**.

**Protein**	**PDB code**	**Disulf**	**χ_1_**	**χ_2_**	**χ_3_**	**χ_2_'**	**χ_1_'**	**Torsion energy kJ/mol**	**Conf**.	**Motif**
AKT	1unr	60–77	−52.3	−120.5	105.2	−82.6	−50.9	13.9	Staple	CSD
ERO1	3ahq	35–48	−54.3	−121.9	45.8	75.6	176.7	36.9	*cis*	CSD
ERO1	3ahq	37–46	−61.3	−79.9	110.1	−102.8	−58.8	11.7	Staple	CSD
CD4	4h8w	130–159	−67.0	−86.3	108.2	−91.1	−58.6	11.7	Staple	CSD
+BASI	1ava	144–148	−63.2	−93.9	115.6	−78.1	−71.0	12.5	Staple	CSD
−BASI	1ava	43–90	−60.1	−73.5	−89.3	−73.6	−67.0	1.6	Spiral	–
+DsbD	1jpe	103–109	−51.0	−116.4	92.1	−77.8	−72.8	13.8	Staple	CSD

Finally, we attempted to assess the likelihood that the reduced form of the protein of interest was a likely target of ROS/RNS; and which of the Cys was more likely to be modified. This was only possible for the PH domain of AKT. The controls we used were reduced forms of Trx-like enzymes and other proteins known to be modified by GSH. The results are shown in Table 2. The pKas of the active Cys in Grxs were generally between 7 and 8, while Cys in proteins which are modified by GSH were between 10 and 11. The values calculated for AKT were in the same range as GSH substrates. From the point of view of pKa, both Cys are equally likely to be attacked by ROS/RNS. However, other considerations may be important *in vivo* such as solvent accessibility of the individual Cys i.e., whether it is buried or accessible.

**Table 2:** **Calculated pKas for AKT and control proteins**.

**Protein**	**Cys**	**PDB**	**pKa**
ScGrx6	136	3l4n	7.42
StGrx2	9^*^	3ir4	7.11
HsGrx2	37	2fls	8.01
HsCLIC1	24	1k0n	9.43
SpGST	10^*^	1f2e	11.56 ± 0.24
PmGST	10^*^	1pmt	11.07
BxGST	10	2dsa	17.29 ± 0.05
HsGSHR	58^*^	1dnc	9.52 ± 0.11
TttRNA methyltransferase	223	3g5s	10.09
Rncarbonic anhydrase	183^*^	1flj	10.57
AKT	60	1unq	11.35
AKT	77	1unq	11.03

* *Indicates the proteins where GSH was removed from Cys for calculation. Standard error was calculated where required*.

## Discussion

Disulfide bonds between Cys residues are generally thought to confer extra rigidity and stability to their resident protein, forming a type of proteinaceous spot weld. The conundrum of disulfides as structural stabilizers is how the two Cys residues could have been introduced into the protein chain simultaneously. A possible explanation is suggested by the emerging paradigm that the disulfide proteome consists of two subproteomes: a structural group and a redox-sensitive group (Yang et al., 2007). For structural disulfides, both Cys are functionally required. However, a single Cys residue can form a redox-sensitive site on a protein. Thus, a redox-active disulfide may be introduced into a protein structure by stepwise mutation of two residues in the native sequence to Cys. By extension, evolutionary acquisition of structural disulfides in proteins can potentially occur via transition through a redox-active disulfide state.

Alternatively, a fully-fledged disulfide of either type could be acquired via a retrotransposon. Indeed, introduction of another type of redox site, the four Cys zinc (Zn^2+^) binding site (Wouters et al., 2010), into proteins appears to largely have been effected by retrotransposons (Babu et al., 2006). A hallmark of this type of acquisition is insertion of a sequence fragment. In the case of Zn fingers, the fragment is around 30 amino acids in length.

The cases of CSD acquisition here clearly do not involve insertion of a sequence fragment, as evidenced by the sequence alignment. Instead, the redox switch was introduced by stepwise mutation of two residues in the native sequence to Cys. The advantages of acquisition of the CSD to protein function are clearly demonstrated by the retention of the redox switch in higher organisms. It is interesting to note that triplication of the AKT gene occurred after acquisition of the CSD, and it remained encoded in all daughter genes, suggesting it is integral to the function of these genes. In some species or homologs, the CSD acquisition process appears to have reversed. For example, in ERO1B, one of the CSDs was lost in secretory tissues of land animals.

**Figure 6:**
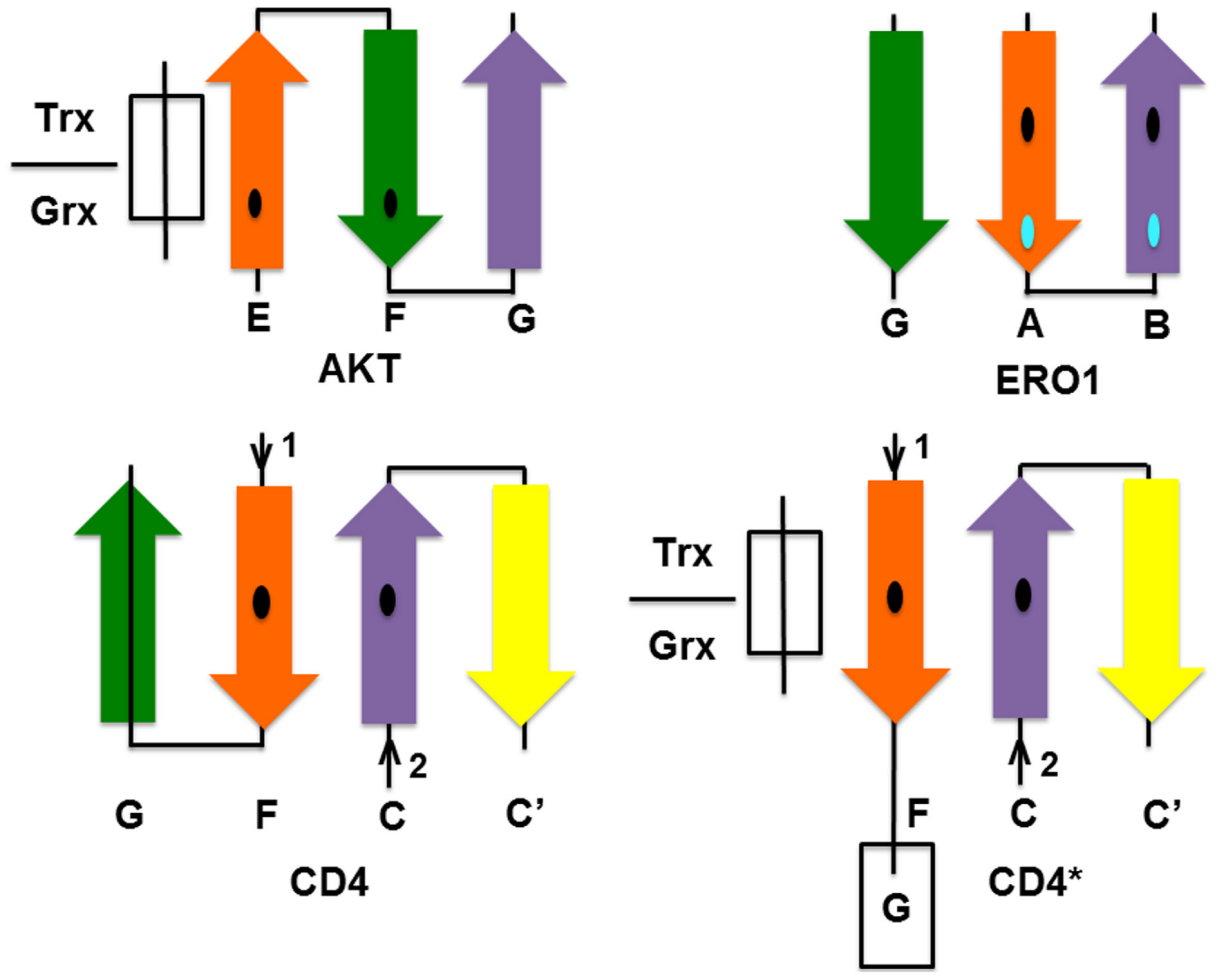
**Location of CSDs in the secondary structure of the proteins studied according to PDB structures: CD4—4h8w, ERO1—3ahq, and AKT—1unr**. The Cys on the orange strand is the putative active Cys for each protein. Trx/Grx must bind as an extra strand near the active Cys as shown for AKT. In ERO1 and CD4, this site is protected by an additional strand (strand G in both CD4 and ERO1), which must be displaced to allow access by Trx. For CD4 to become accessible to Trx/Grx, one of the hairpins must hinge open to allow Trx/Grx to dock in place of the strand as shown in the hypothetical CD4^*^ diagram.

Do the two Cys need to be acquired in a particular order during the evolutionary process? In order to be glutathionylated, a minimum requirement is that the Cys has a low pKa. When two disulfide-forming Cys are present, the Cys with the lower pKa is generally termed the “active” Cys (Figure 1), because it is the one targeted by ROS/RNS. The second Cys, which prevents overoxidation of the active Cys by disulfide formation, is termed the “resolving Cys.” However, the pKas of the two Cys are not easily discerned from a protein structure and are generally determined experimentally. In addition, the structural requirements of Grx and Trx docking are such that the active Cys should be on an edge strand (Figure 6). Thus, by the simplistic hypothesis depicted in Figure 1, the low pKa Cys should be introduced into the protein first and be mutated from the protein last; and it should be on an edge strand. How does this hypothesis stack up with the three proteins studied here? The relevant information is summarized in Table 3.

**Table 3:** **Contexts of CSD residues in proteins of interest**.

**Protein**	**N-Cys**	**Edge?**	**C-Cys**	**Edge?**	**Environment**
CD4	130	No	**159**	No	Cell surface
ERO1	**35**	No	48	Yes	ER
ERO1	**37**	No	46	Yes	ER
AKT	**60**	Yes	77	No	Cystosol

*The table is based on PDB structures 1unr (AKT), 4h8w (CD4), and 3ahq (ERO1). The most common 1-Cys residue in the sequence alignments is bolded. This is likely to be the “active” Cys*.

Of the three molecules, only AKT can dock Grx/Trx in the manner suggested, as shown in Figure 6. However, of the three molecules, AKT is the only cytosolic protein and thus would be found in the reduced state under normal physiologic conditions. Thus, consideration of the cellular compartment is an additional complication. Oxidation of a cytosolic molecule, promoting formation of the disulfide-bonded form, will only occur under conditions of oxidative stress. Disulfide bond formation in the pleckstrin-homology domain of AKT is likely associated with membrane targeting of AKT under conditions of oxidative stress (Fan et al., 2009). The likelihood that Cys 60 is the active Cys of the AKT CSD is supported by studies which demonstrate its modification in experiments on growth factor signaling (Antico Arciuch et al., 2009). On the other hand, CD4 and ERO1 exist in oxidative environments where the disulfide bond would be formed in the latent state: CD4 is cell surface protein of the blood plasma; ERO1 is an ER-resident protein.

Only in the cytosolic protein AKT, where the disulfide is normally in the reduced state, is the protein readily accessible by Trx. In CD4 and ERO1, the putative Trx-binding site is blocked by an additional strand. In CD4, the additional strand G is formed by residues 166–172 (PDB: 4h8w). In ERO1, strand G is formed by residues 355–359. Protection by an additional strand may be a general feature of CSDs in oxidative environments to prevent adventitious reduction of the CSD.

Because the disulfides in ERO1 and CD4 are latently oxidized, it is likely that Trx is only given access to the site under special conditions. For example, in CD4 the site becomes accessible during T-cell signaling. It has been proposed that CD4 undergoes domain swapping to facilitate this reduction (Matthias et al., 2002), a process that involves rearrangement of β strands. We speculate on the nature of this process in CD4 below. The latently oxidized states, are thus likely “occult” Trx-binding sites which must be unmasked by conformational changes prior to Trx binding and disulfide reduction (Wouters et al., 2010).

Several other proteins containing CSDs domain swap suggesting a relationship between domain swapping and CSD reduction (Wouters et al., 2010). GSH binding may also be related to domain swapping: GSH binding to Cys 60 of glyoxalase I regulates domain swapping in this protein (Saint-Jean et al., 1998).

### Evolution of CD4

Several evolutionary studies of CD4 have examined the non-synonymous/synonymous substitution rate (ω = dN/dS) in order to determine which regions of the CD4 molecule are under positive selection. Non-synonymous changes are nucleotide codon changes that alter the amino acid coded at the protein level, while synonymous changes alter the nucleotide while preserving the amino acid. These studies found that CD4 is evolving rapidly under positive selection. In a pairwise comparison of human and mouse CD4, Ansari-Lari et al. (1998) determined ω = 0.77, much higher than the average of ω = 0.12 determined for ~13000 pairs of human/mouse orthologs. A study of primate CD4 sequences identified positive selection (ω > 1) throughout the molecule, but chiefly in the N-terminal region corresponding to D1 and D2 (Zhang et al., 2008). The sequence up to residue ~153, which includes the first Cys residue of the CSD, has stronger selection than average for the molecule (ω > ω^−^ = 1.17).

Our study shows that the relatively rapid evolution of CD4 is predominantly focused on acquisition and modification of thiols involved in redox signaling. CD4 is an interesting molecule from the point of view of evolution of thiol-based redox signaling. All three known protein-protein interaction sites contain important cysteine residues. The primordial cysteine site: the CXC motif found in the cytoplasmic tail, is not found in the ancestral gene LAG3. This motif mediates the interaction with p56LCK necessary for T cell activation (Laing et al., 2006).

A second site is the CSD in D2, which is the subject of this study. This is the primary recognition site for HIV gp120 and a co-recognition site for MHCII molecules. The independent acquisition of the CSD in two clades, primates and muroids, at roughly the same epoch ~75 MYA is interesting, and could arise from an environmental change, for example in the oxygenation of the Earth's atmosphere. This date is not long after the KT extinction, the global event that wiped out the dinosaurs around 80 MYA. Alternatively the dual acquisition might have a biological origin e.g., challenge by a common parasite or infectious agent. Mammals evolved during an interesting period of the earth's history with respect to continental drift. During the creataceous period, the super-continent Gondwana broke up. Given the co-location of the two clades involved on the same continent, the second “biological challenge” hypothesis seems attractive. The pairwise sequence alignments show the CSD site has been undergoing rapid evolution since its introduction in both clades (Figure S2).

The third protein-protein interaction site bearing important Cys residues is located in D1. Interestingly, in a process similar to step 1 of the CSD evolution in D2, modification of the canonical Ig disulfide between strands B and F has also occurred in D1 of some fish CD4 proteins (trout and fugu), which contain a distinct unpaired Cys in strand F, but lack the second Cys in strand B that forms a disulfide in mammalian and bird CD4 molecules.

Finally, LAG3 is very similar to CD4 but lacks the LCK binding site (Laing et al., 2006) (α in Figure 2B). Functionally LAG3 interacts with MHCII with higher affinities than CD4, and may have a role in impeding access to MHCII of CD4 (Workman et al., 2002; Workman and Vignali, 2003). The two Ig-domain molecule CD4REL (β in Figure 2B) appears to be a progenitor of CD4 which is also found in the lamprey a vertebrate which diverged earlier than fish. The lamprey genome does not contain a CD4 ortholog (γ in Figure 2B). Trout contains both CD4REL and CD4, but vertebrates that evolved later, such as the chicken, do not appear to have a CD4REL-like molecule, retaining only CD4.

Thus, CD4 arose *de novo* in fish around 450 MYA around the time its primary tissue of origin, the thymus, appeared in animals. Originally containing the important cytosolic CXC motif, CD4 then underwent rapid evolution at two distinct protein-protein interaction sites in D1 and D2, both bearing important Cys residues.

#### Speculations on activation of CD4 by HIV gp120

Given the importance of CD4 CSD reduction in the entry of HIV gp120 into the cell, it is worth speculating on the nature of this process in the light of the hypothesis put forward here. Sheet 1 in D2 of CD4, which consists of residues 99–119 and 142–146, can be considered as the “torso” of D2 from which two β-hairpin “arms” are extended, comprising residues 127–140 (N-arm) and residues 157–172 (C-arm). These “arms” are clasped together by a series of hydrogen bonds and the disulfide formed between Cys 130 and Cys 159 on strands C and F to form the second sheet. In domain swapping, either one or both of these arms may be exchanged with an adjacent CD4 molecule. This can only be achieved by breakage of the C-F hydrogen bonds and reduction of the CSD between Cys 130 and Cys 159. In its location on the interior strands of the four-stranded sheet 2, the CSD is protected from interaction with Trx and Grx. However, it may be accessible by GSH. In order to become accessible to Trx/Grx, one of the hairpins must hinge open, to allow Trx/Grx to dock in place of the strand. As Cys 159 is conserved in all CD4 molecules, it is likely the “active Cys.” Thus, docking of Trx/Grx may be effected by peeling away the C-terminal strand (G) from the molecule (CD4^*^ in Figure 6). Thus, we propose this is a necessary “priming” step for CD4 CSD reduction.

### Context-dependence of Cys incorporation in protein sequences

Early studies on protein sequences indicated Cys is more abundant in more complex organisms (Fahey et al., 1977; Miseta and Csutora, 2000). This has been confirmed by more recent studies on whole genomes that have shown the abundance of Cys in proteins has been increasing since divergence from the Last Universal Common Ancestor (Woese, 1998; Brooks and Fresco, 2002); and this increase is a continuing panspecies phenomenon which is evident within the last 10 million years (Jordan et al., 2005). It seems likely that increased Cys abundance arises from increased use of thiol-based redox signaling in more complex organisms. Although the prevalence of Cys in proteins is increasing, the abundance has not yet reached the neutral frequency (i.e., the frequency that would be expected based on the number of Cys codons in the genetic code) (King and Jukes, 1969; Brooks and Fresco, 2002). The failure of Cys to reach its equilibrium frequency suggests that incorporation of additional Cys into proteins may come at a cost: increased signaling and control may be gained at the cost of deleterious effects of over-oxidation (Wouters et al., 2010).

This study suggests that successful incorporation of Cys may be limited to very select contexts in proteins, specifically those where reduction of the Cys can be effected by enzymes of redox homeostasis. In this study, the successfully incorporated sites are likely reduced by Trx-like enzymes. The disulfide bond in CD4 is reduced by Trx (Aniksztejn et al., 1987; Matthias et al., 2002), and the disulfide in AKT may be sensitive to Grx (Murata et al., 2003; Wang et al., 2007).

If a Cys that is modifiable by GSH is in a place which cannot be accessed by Grx, the protein will likely be targeted for removal by the GSH detoxification system. Thus, the mutation most likely will have a deleterious effect on protein function by inducing a state of haploinsufficiency. If the Cys is in a place where it may be reduced by Grx, introduction of the Cys does not have a deleterious effect on protein function because it may be removed. If, in addition, it is introduced in a place which modulates the protein's function in a way which is beneficial to redox function, it may become fixed in the population. For example, if addition of GSH temporarily prevents a protein interaction that would be deleterious under conditions of oxidative stress. Thus, only 1-Cys GSH switches that are beneficial to physiologic function are likely to be retained in the population sufficiently long for random mutation of a second Cys to occur; and this second mutation must also be sufficiently beneficial to be retained over the 1-Cys variant.

### Convergent acquisition of modular redox switches

Before multiple genome sequences were complete, it was generally believed increased complexity of organisms correlated with gene number. After completion of the first genomes, the small differences in gene number between simple unicellular eukaryotes and mammals forced revision of how complexity is encoded. For example, the yeast genome contains 5000 genes, yet the human genome contains only five times more: 25,000. Additional complexity at the organismal level is likely encoded at the molecular level by noncoding DNA, as this is considerably different between these organisms (Mattick, 2004).

However, increased complexity may also be encoded at the protein level. It was previously recognized that concatenation of existing domains through gene fusion, also known as protein domain mosaicism, encodes new functions in more complex organisms (Patthy, 1985). Studies on the changing amino acid content of proteins show that domains also are not static structures. Additional complexity added to protein domains in the form of redox and other switches likely increases the signaling capabilities of individual domains. In other words, nature is continually tinkering with these independent folding units: a domain from archaea may not have the same sophisticated set of switches as the homologous domain from a mammalian protein.

Thus, two modes of acquisition of increased protein complexity have been demonstrated to date: protein domain mosaicism (Patthy, 1985), and acquisition of allosteric control sites. Previously, Babu et al. (2006) showed that Zn finger allosteric control sites are added to protein sequences via retrotransposons. Here we studied a novel method of acquisition of an allosteric control site: formation of CSDs by stepwise acquisition of Cys residues in appropriate contexts. Both these Cys-based sites, Zn fingers and CSDs, are known to be redox regulated.

In this study, we analyzed incorporation of disulfide-based redox switches into three protein families. Successful context-dependent stepwise evolution of the sites likely enabled the proteins to utilize existing Trx-like enzymes to mediate successful incorporation into cellular mechanisms of redox homeostasis. Thus, three non-homologous proteins have convergently acquired the same modular redox switch: the CSD. CSDs are, by far, the most common forbidden disulfide found in homologous sites of protein structures (Wouters et al., 2010). They have evolved independently multiple times. CSDs have been postulated to have special redox properties: specifically intermediate torsional energies that enable them to be reduced in a controlled process by other thiol sites in proteins. However, many other forbidden disulfides share this property, so this does not explain why evolution of CSDs is favored over other forbidden disulfides. Work by Maeda et al. (2005), which suggests that a CSD in BASI is specifically recognized by Trx; and our own bioinformatic work, which has identified multiple CSDs which undergo redox reactions with the CXXC motif of Trx-like enzymes, suggest that CSDs are cognate substrates of Trx-like enzymes (Haworth and Wouters, 2013). Although Trxs recognize multiple non-homologous substrates, solved protein structures of disulfide-linked Trx-substrate reaction intermediate mimics have provided evidence for recognition of structural motifs in target proteins by Trxs (Qin et al., 1995, 1996; Maeda et al., 2006; Chartron et al., 2007).

Although BASI has nine disulfides, only two are specifically targeted by Trxh (Maeda et al., 2005). Cys 144–Cys 148 and Cys 43–Cys 90. Of these two, Cys 144–Cys 148, which is a CSD, is much more efficiently reduced. The specificity of the reaction is partly ensured by consistent docking of the substrate. In most solved protein structure complexes, Trx-like molecules bind their substrates as an antiparallel β-strand. The interaction between Trx and its substrate is characterized by three distinctive hydrogen bonds of a pattern typically found between pairs of antiparallel β-strands. A similar pattern of hydrogen bonds is observed between the N-terminal Trx-like domain of *Escherichia coli* DsbD in complex with the C-terminal domain of DsbD which contains the CSD (Cys 103–Cys 109) substrate (Rozhkova et al., 2004). This suggests the evolution of CSDs is favored over other forbidden disulfides because they can be immediately exapted from the Grx to the Trx redox homeostasis pathways.

### Conflict of interest statement

The authors declare that the research was conducted in the absence of any commercial or financial relationships that could be construed as a potential conflict of interest.

## Footnotes

^1^http://www.sbinf.org/applications/pes.html

^2^http://www.pymol.org/

^3^http://propka.org
