# Supplementary material for: Potential role of glutathione in evolution of thiol-based redox signaling sites in proteins
Source: Front Pharmacol. 2015 Mar 10;6:1. doi: 10.3389/fphar.2015.00001 (PMC4354306; doi:10.3389/fphar.2015.00001)
Supplement: Supplementary file 1 [file DataSheet1.PDF]

20/12/2014

Supplementary Material

Potential role of glutathione in evolution of thiol-based redox signaling sites in proteins

Kaavya A Mohanasundaram$_1$[1], Naomi L Haworth, Mani P Grover$_3$[1], Tamsyn Crowley$_4$[1,2], Andrzej Goscinski$_5$[3], Merridee A Wouters$_6$[1*]

[1]School of Medicine, Faculty of Health, Deakin University, Geelong, Victoria, Australia
[2]Australian Animal Health Laboratory, CSIRO Animal, Food and Health Sciences, Geelong, Victoria, Australia
[3]School of Information Technology, Faculty of Science, Engineering and Built Environment, Deakin University, Geelong, Victoria, Australia

*** Correspondence:** Merridee A Wouters, School of Medicine, Faculty of Health, Deakin University, Geelong, Victoria, 3216, Australia.
m.wouters@deakin.edu.au

1. Supplementary Figures

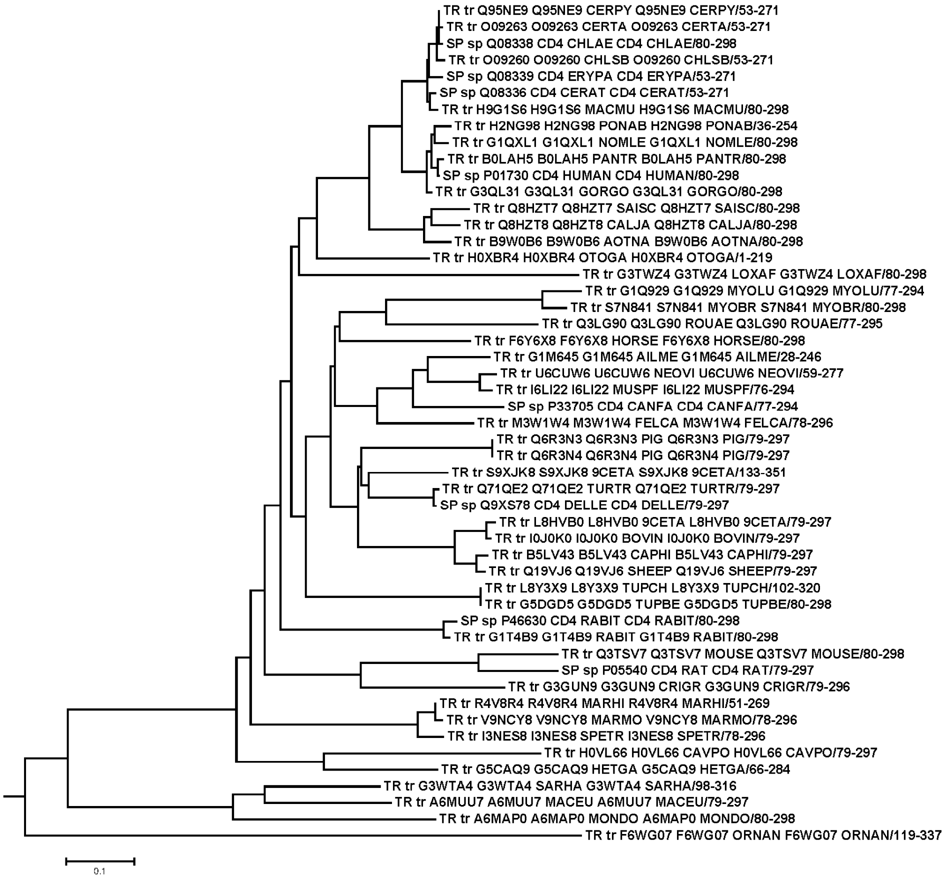

Supplementary Figure 1. Phylogram of 51 CD4 sequences

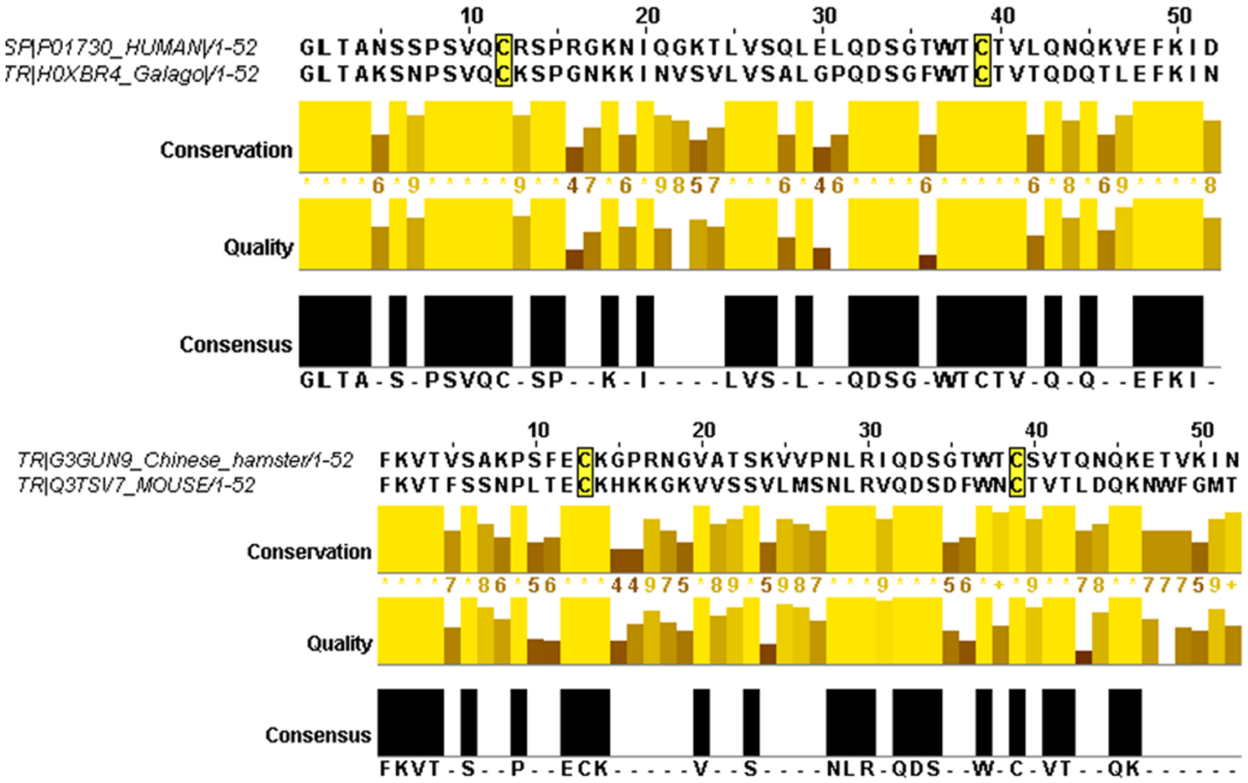

Supplementary Figure 2. Pairwise alignment of CD4 sequences (a) in the region of the CSD between human & galago (upper panel) and mouse & Chinese hamster (lower panel).

(a)

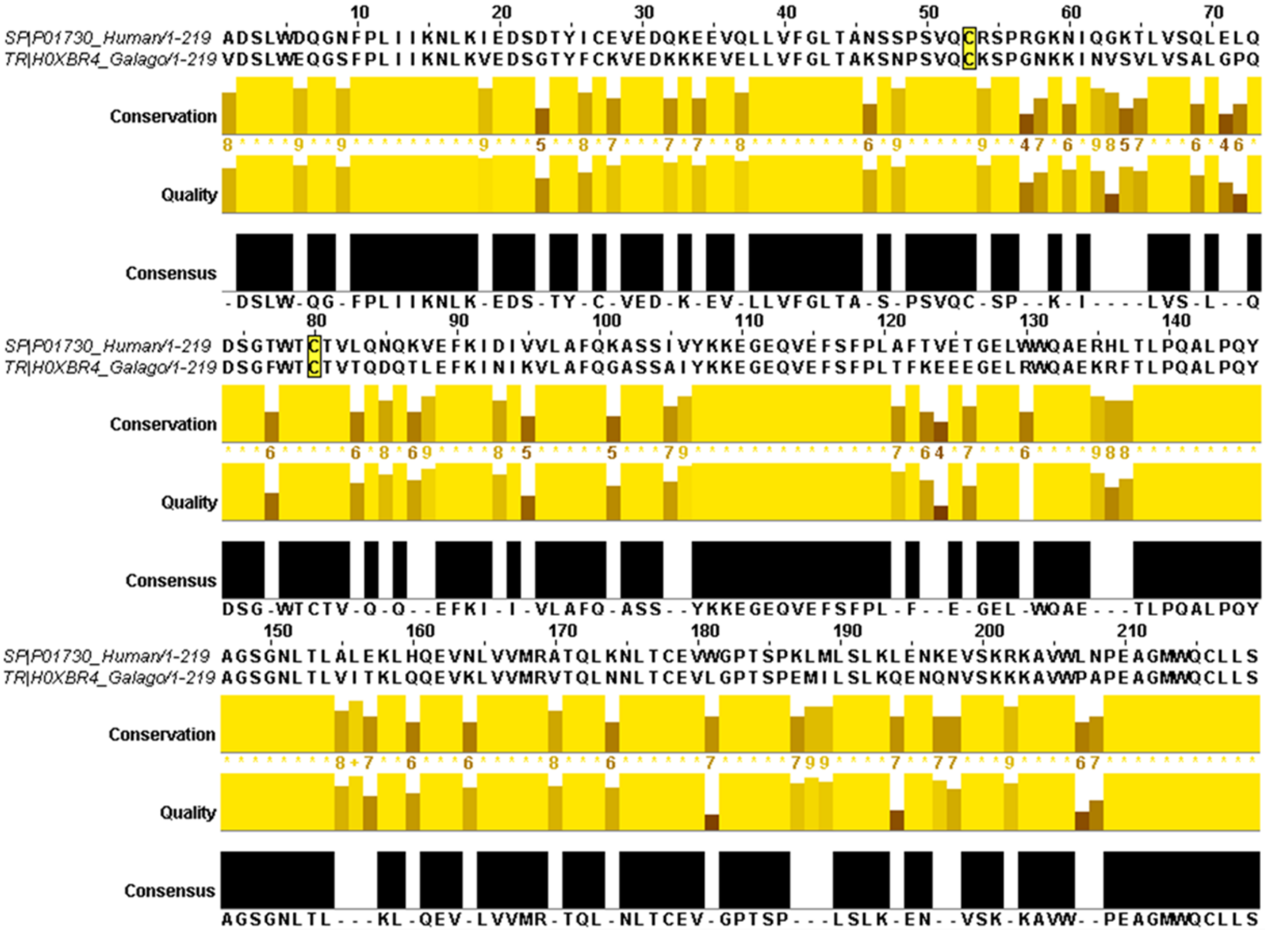

Supplementary Figure 2 cont. Pairwise alignment of CD4 sequences (b) between human and galago over the full length sequence

(b)

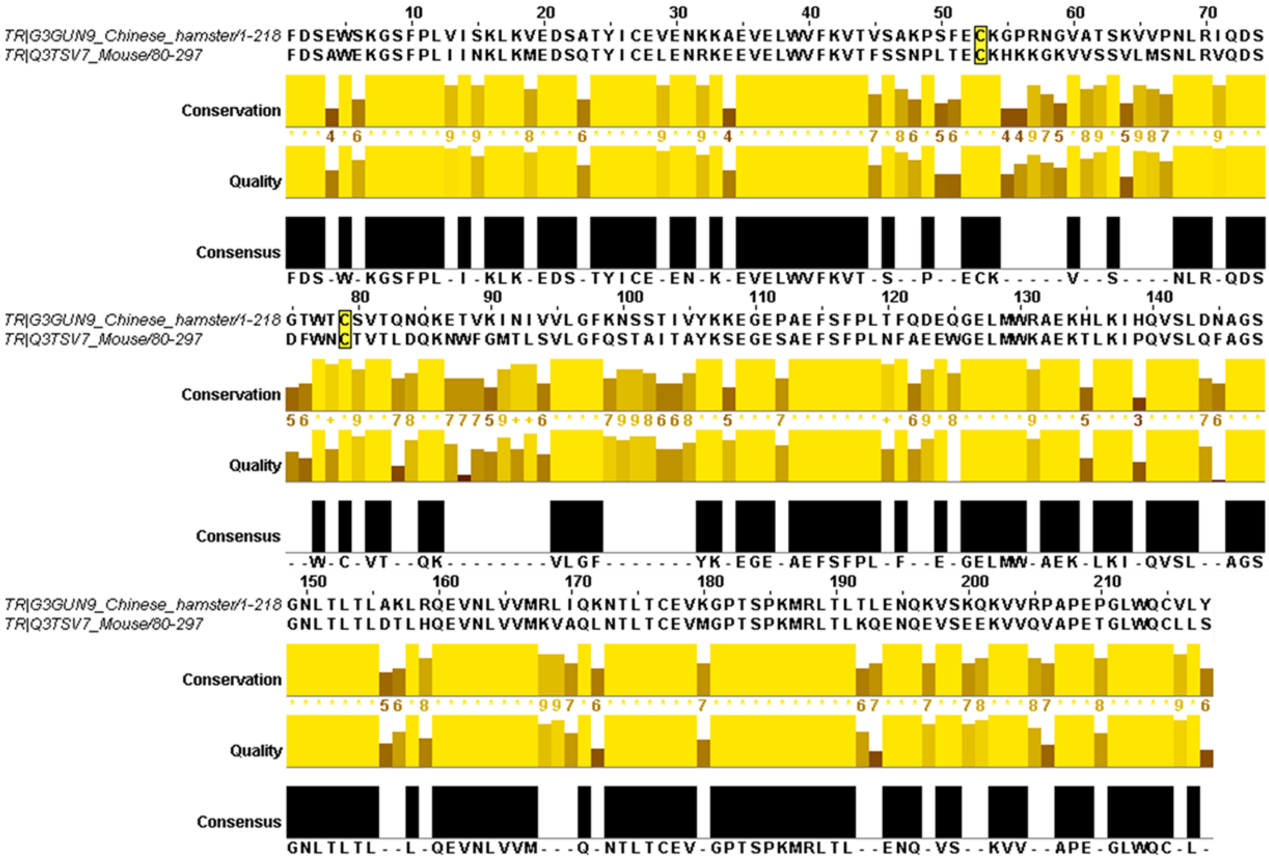

(c)

Supplementary Figure 2 cont. Pairwise alignment of CD4 sequences (b) between mouse and Chinese hamster over the full length sequence

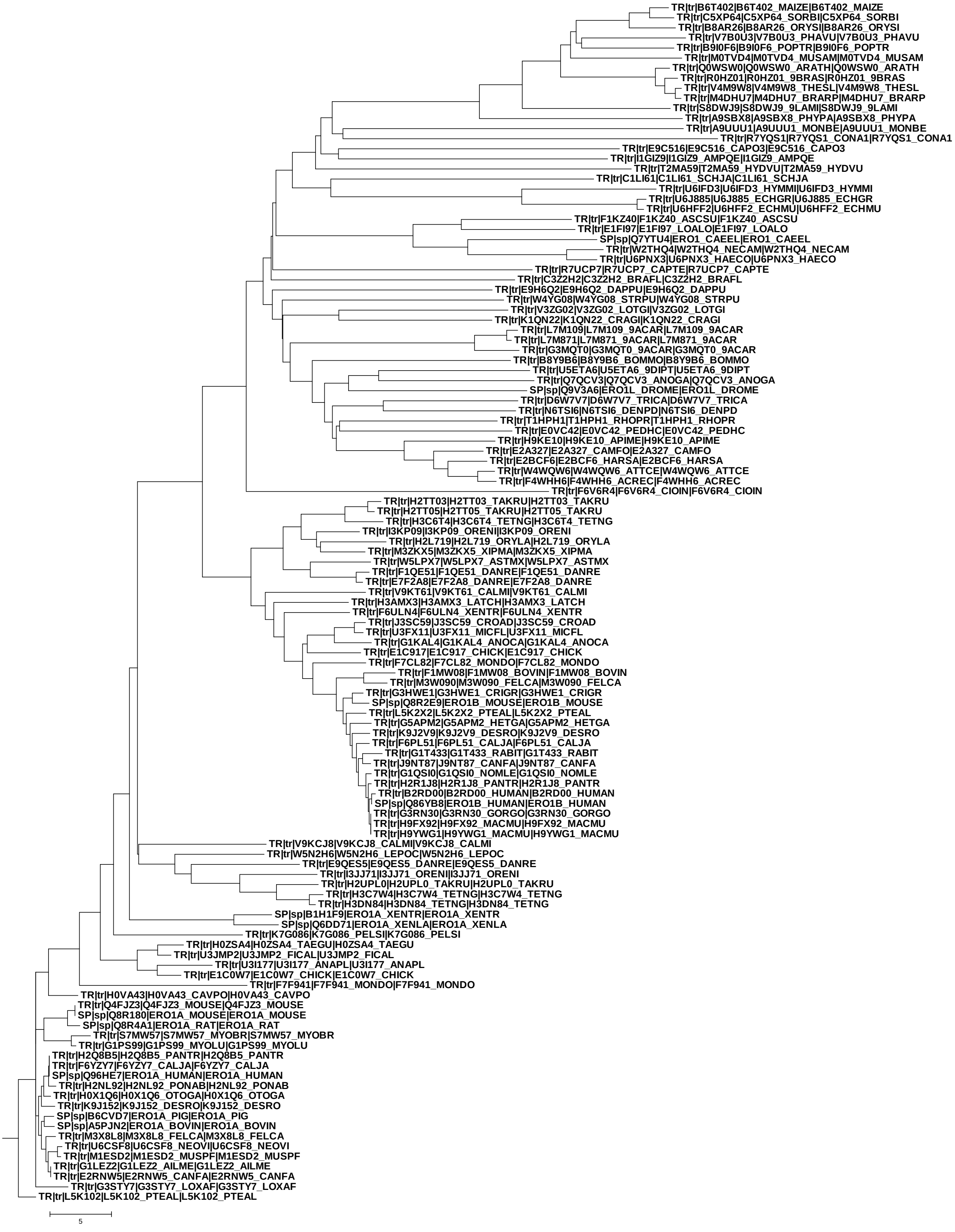

Supplementary Figure 3 Phylogram of 119 ERO1 sequences

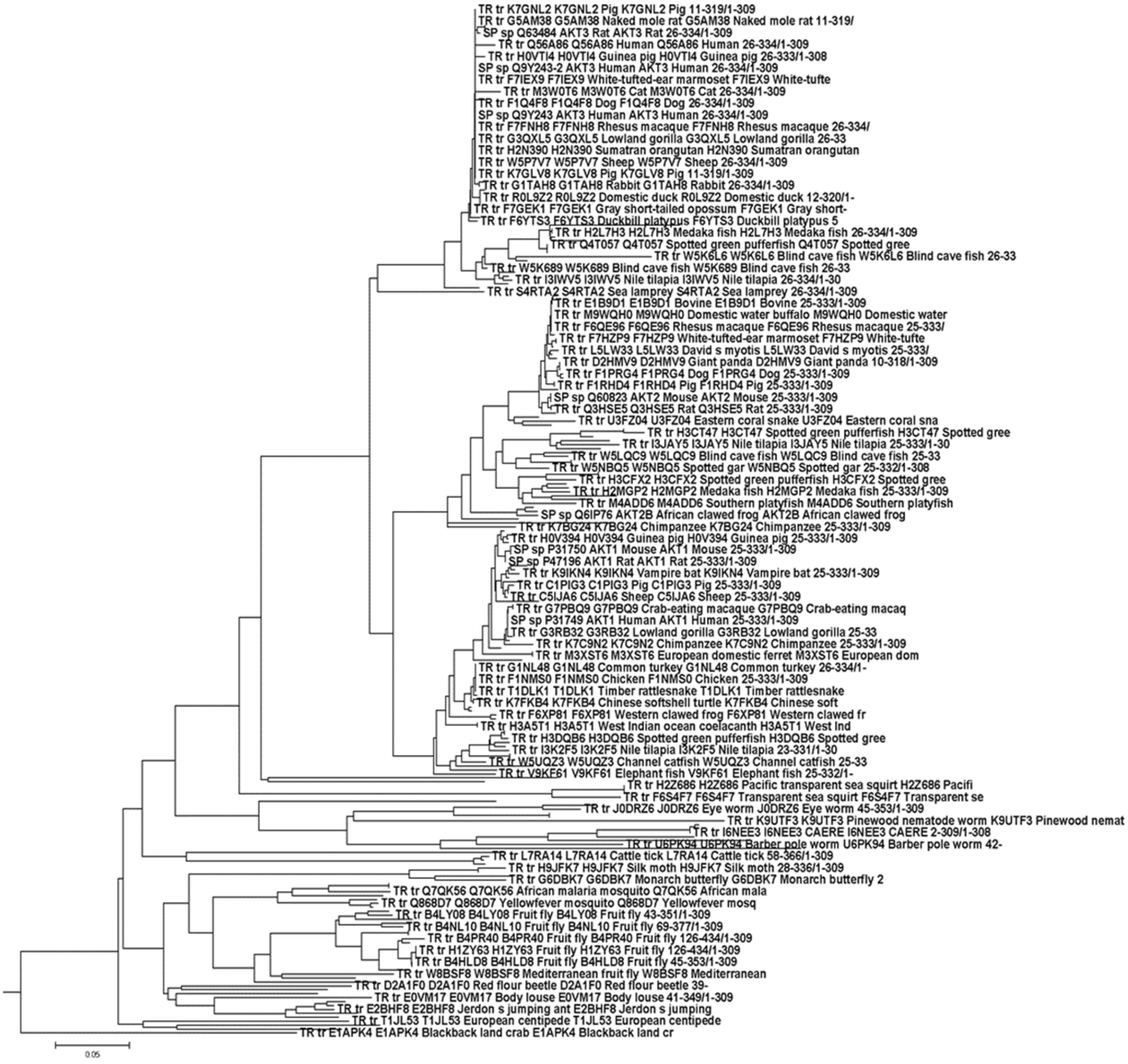

Supplementary Figure 4 Phylogram of 262 AKT sequences
